# Ravulizumab for generalized Myasthenia Gravis: a multicenter real-life experience

**DOI:** 10.1007/s00415-025-13127-8

**Published:** 2025-05-14

**Authors:** Elena Rossini, Vincenzo Di Stefano, Raffaele Iorio, Francesco Habetswallner, Michelangelo Maestri, Claudia Vinciguerra, Elena Maria Pennisi, Giuseppe Di Martino, Nicasio Rini, Silvia Falso, Sofia Marini, Dario Ricciardi, Melania Guida, Stefania Morino, Matteo Garibaldi, Luca Leonardi, Demetrio Marando, Laura Tufano, Giovanni Antonini, Laura Fionda

**Affiliations:** 1 Department of Neuroscience, Mental Health and Sensory Organs (NESMOS), Faculty of Medicine and Psychology, SAPIENZA University of Rome, Sant’Andrea Hospital, Via Di Grottarossa 1035-1039, 00189 Rome, Italy; 2 Neuromuscular and Rare Disease Centre, Neurology Unit, Sant’Andrea Hospital, Rome, Italy; 3 Biomedicine, Neuroscience and Advanced Diagnostic (BIND) Department, University of Palermo, Palermo, Italy; 4 Department of Neuroscience, Università Cattolica del Sacro Cuore, Rome, Italy; 5 Neurology Unit, Fondazione Policlinico Universitario Agostino Gemelli IRCCS, Rome, Italy; 6 UOC Neurophysiopathology, AORN Cardarelli, Via Antonio Cardarelli 9, 80131 Naples, Italy; 7 Department of Clinical & Experimental Medicine, Neurology Unit, University of Pisa, 56126 Pisa, Italy; 8 Department of Medicine Surgery and Dentistry, Neurology Unit, University Hospital “San Giovanni Di Dio E Ruggi d’Aragona”, Scuola Medica Salernitana”, Neuroscience Section, Salerno, Italy; 9 Neuromuscular Diseases Center, Neurology Unit, San Filippo Neri Hospital, Rome, Italy; 10 UniCamillus-Saint Camillus International University of Health Sciences, Rome, Italy

**Keywords:** Myasthenia Gravis, Complement inhibitor therapy, Ravulizumab, Real life study

## Abstract

**Introduction:**

Ravulizumab, a monoclonal antibody targeting C5, was recently approved for the treatment of anti-AChR positive generalized myasthenia gravis (gMG) patients. The objective of this study is to present the Italian multicenter real-world experience evaluating the safety and efficacy of ravulizumab in gMG within the context of the Expanded Early Access Program (EAP).

**Methods:**

We conducted a retrospective study in 7 gMG referral centres in Italy. Demographic and clinical characteristics were recorded at baseline and during follow-up through clinical scale changes including Myasthenia Gravis-Activities of Daily Living (MG-ADL), Quantitative Myasthenia Gravis (QMG) and Myasthenia Gravis Composite (MGC). Frequency of minimal symptom expression (MSE) and changes in concomitant medications were also evaluated.

**Results:**

Twenty-four gMG patients (10/24 females) aged between 24 and 82 years (Median 60.5, IQR 52.5–67.5), were included. Fifteen patients had undergone thymectomy, and 14 had a thymoma. Median follow-up duration was 26 weeks (range 10–74, IQR 26–42). MG-ADL and QMG scores showed a significant decrease with respect to baseline (*p* < 0.001). MSE was achieved by 37.5% patients at the last available follow-up. Tapering of prednisone daily dosage was possible in 76% of patients. Thymoma was significantly associated with QMG score reduction and the frequency of QMG responders at week 2 (*p* = 0.03).

Three patients discontinued treatment. One patient experienced a myasthenic exacerbation and needed rescue therapy. Infectious adverse events were reported in 5/24 patients, and a Stevens-Johnson syndrome in one patient.

**Conclusions:**

Real-world data confirm the effectiveness, safety, and prednisone-sparing effect of ravulizumab in patients with gMG, especially in those with thymoma.

## Introduction

Myasthenia Gravis (MG) is an autoimmune disorder characterized by muscle weakness and fatigability, resulting from the presence of autoantibodies that target components of the neuromuscular junction (NMJ) [1]. In approximately 80% of patients, these antibodies are directed against the acetylcholine receptor (AChR). The predominant subclasses of these antibodies are IgG1 and IgG3, which mediate their effects through multiple mechanisms: blocking acetylcholine from binding to its receptor, inducing the internalization of AChRs via autoantibody-mediated crosslinking, and activating the complement cascade, thereby causing tissue damage at the NMJ [2, 3]

The classical complement pathway is activated when C1q binds to antigen–antibody complexes formed by the binding of IgG1 or IgG3 autoantibodies to AChRs. This interaction triggers the complement cascade, leading to the cleavage of C3 into C3a and C3b, and of C5 into C5a and C5b. Ultimately, this cascade culminates in the formation of membrane attack complexes (MACs), which cause localized lysis of the post-synaptic membrane. This process results in the destruction of the post-junctional folds and a failure of neuromuscular transmission [4]. MAC deposition is essential for complement-mediated endplate damage, and serum complement levels are inversely correlated with the clinical severity of generalized MG (gMG) [5].

On these bases, various complement inhibitors have been evaluated in clinical trials over the past decade and have demonstrated efficacy in the treatment of gMG. Following the approval of eculizumab and zilucoplan, the FDA approved in April 2022 for ravulizumab, a humanized IgG2/4κ monoclonal antibody that specifically binds to complement protein C5, inhibiting its cleavage into C5a and C5b. Ravulizumab efficacy and safety were assessed in a phase 3, randomized, double-blind, placebo-controlled trial (CHAMPION MG; ClinicalTrials.gov identifier: NCT03920293; EudraCT number: 2018–003243-39). This study demonstrated rapid and sustained improvements in both patient- and clinician-reported outcomes, with a manageable adverse event profile [6, 7]. However, the patient population in the CHAMPION MG trial had specific clinical characteristics, and long-term real-world evidence remains limited.

The aim of this study is to present an Italian real-world experience involving patients with anti-AChR-positive gMG treated with ravulizumab, within the framework of the Expanded Early Access Program (EAP) initiated in Italy in March 2023.

## Patients and methods

### Study design and inclusion criteria

We conducted a multicentre, observational, retrospective study to evaluate the safety and efficacy of ravulizumab in patients with gMG in a real-world clinical setting. Seven Italian MG referral centres participated in the study: Sant'Andrea Hospital (Rome), Policlinico Universitario "Paolo Giaccone" (Palermo), Fondazione Policlinico Universitario Agostino Gemelli IRCCS (Rome), Cardarelli Hospital (Naples), Azienda Ospedaliera Universitaria Pisana (Pisa), San Giovanni di Dio Ruggi d’Aragona (Salerno), and San Filippo Neri Hospital (Rome).

The study was conducted as part of the EAP, which aimed to provide eligible gMG patients with access to ravulizumab treatment before its regulatory approval.

Patients were considered eligible for inclusion if they met the following criteria:

(i) Treatment with ravulizumab as part of clinical practice started within the EAP program with a follow-up of at least ten weeks (i.e., after the third infusion).
(ii) Age ≥ 18 years at the time of informed consent and treatment initiation.
(iii) Myasthenia Gravis Foundation of America (MGFA) classification IIb, IIIa, IIIb, IVa, IVb, V at baseline.
(iv) Myasthenia Gravis Activities of Daily Living (MG-ADL) ≥ 6 at baseline. [8]
(v) Post-Intervention Status Unchanged or Worsened after treatment with corticosteroids and at least and no more than one other non-steroid immunosuppressant (NSIST), administered at adequate dosages and for an adequate duration, but with persistent symptoms or side effects that impaired functionality, as assessed by both the patient and the treating physician, in line with the requirements of the EAP.

Patients previously treated with eculizumab, as well as those previously enrolled in CHAMPION trial, were excluded.

### Treatment schedule and vaccinations

Ravulizumab was administered intravenously according to the patient's weight, with an initial loading dose of 2400 mg, 2700 mg, or 3000 mg. This was followed by a second infusion two weeks later, consisting of a maintenance dose of 3000 mg, 3300 mg, or 3600 mg. Subsequent infusions were administered every 8 weeks ± 7 days (Weeks 10, 18, 26, etc.) throughout the duration of the study.

All ravulizumab infusions were provided in an outpatient setting at the respective reference hospital.

Before ravulizumab start, all patients were vaccinated against *Neisseria meningitidis* serogroups A, C, W135, Y, and B, with vaccination occurring at least 14 days before the first dose of ravulizumab. Alternatively, patients received appropriate prophylactic antibiotics before treatment initiation and continued until the completion of the vaccination regimen.

Ravulizumab was supplied by Alexion as part of the EAP in Italy.

### Data collection

Data were collected retrospectively from medical records at each participating center between September and December 2024. Local databases were used as the primary source for data extraction. A standardized database template, with predefined criteria for data categorization, was distributed across all participating centers. Subsequently, the collected data files were consolidated into a single centralized database and prepared for analysis.

The following variables were included in the data collection: current age, sex, and weight; comorbidities; age at onset of MG and age at treatment initiation; disease duration at baseline; MGFA clinical classification at disease onset and at baseline; history of thymectomy and thymic status; previous treatments, including prednisone, NSIST, and chronic IVIg or PLEX, as well as the number of cycles administered during the year before starting ravulizumab; history of prior myasthenic exacerbations and/or myasthenic crises (MC), and any use of rescue therapies.

Previous and baseline dosages of prednisone and NSIST were recorded. During ravulizumab therapy, any changes in the dosages of ongoing baseline medications, adverse events, treatment discontinuation, and the use of rescue therapies were also recorded.

### Clinical scales and outcome measures

The following clinical scales, specific to gMG were administered by trained clinicians, accordingly to each center clinical practice and EAP indication: (i) MG-ADL (ii) Quantitative Myasthenia Gravis (QMG) [9], and (iii) Myasthenia Gravis Composite (MGC). [10] Data were retrospectively collected at baseline and at each infusion timepoint, up to the most recent follow-up, across all participating centers. Minimal symptom expression (MSE) as defined by MG-ADL ≤ 1 was assessed for each patient at the follow-up end. [11]

Changes in MG-ADL and QMG scores over time were the primary outcome measures of the study to evaluate the efficacy of ravulizumab treatment. Patients were classified as responders if they demonstrated a reduction of at least 3 points in the MG-ADL score (MG-ADL responders) and a reduction of at least 5 points in the QMG score (QMG-responders) at each time point of the follow-up.

MSE achievement was defined in patients who reached MG-ADL score of 0 or 1. Secondary outcome measures included tapering or withdrawal of prednisone and NSIST during follow-up, as well as the comparison of the need for IVIg or PLEX cycles in the year before and during ravulizumab treatment.

Safety was assessed across all centers by monitoring and reporting adverse events or complications throughout the treatment period, and during the follow-up phase for patients who discontinued treatment. This was conducted in accordance with Good Clinical Practice guidelines, which mandate ongoing clinical monitoring—and, when necessary, laboratory and/or radiological assessments— during EAPs, that provide a way for patients with serious diseases to access investigational drugs outside of clinical trials, but still under strict oversight.

### Statistical analysis

We provide a descriptive analysis for demographic characteristics and baseline variables. This included frequency and percentage for categorical variables, median and interquartile range (IQR) or mean ± standard deviation (SD), for continuous variables, unless otherwise stated, e.g. we also calculated 95% CI of the mean to compare our data with those of other studies.

As patients entered the intervention at different times and their follow-up at the time of this study was highly variable, ranging from 2 to 74 weeks (median 26, IQR 26–42), we performed a missing value analysis. [12] The percentage of missing data across the 11 time points of evaluation ranged from 0 to 81.8% for both MG-ADL and QMG and, in total, 125 records out of 264 (47%) were incomplete. Given that up to week 26, the percentage of missing data was less than 21% [5 patients with missing data at week 10 (2 for MG-ADL and 1 for QMG), 18 (2 for MG-ADL and 3 for QMG) and 26 (1 for MG-ADL and 1 for QMG) and 19 patients had no missing data], we evaluated MG-ADL and QMG scores at weeks 2, 10, 18 and 26 for statistical analysis. To determine whether the missing value process was random and the imputation was safe, we used the missing completely at random (MCAR) test (excluding patients who discontinued treatments because of side effects). The comparison between the variance of valid cases and missing value cases across both variables MGADL and QMG at weeks 10, 18 and 26 was non-significant for QMG and significant in comparison of MG-ADL at week 10 and at week 2 (*p* = 0.015). However, the MCAR test was non-significant (*p* = 0.371), indicating the randomness of the missing value process and allowing us to perform data imputation, which we did using the mean method.

Adverse events and exacerbation, as well as eventual use of rescue therapies, have been registered from baseline to week 72.

The presence of outliers and the normality of the distribution of MG-ADL and QMG scores were tested using a boxplot and the Shapiro––Wilk test, respectively. If the variables had no outliers and were normally distributed, a one-way repeated measures ANOVA was performed to determine whether there were statistically significant changes from baseline throughout the intervention. Otherwise, the non-parametric Friedman test with Bonferroni correction for multiple comparisons was used for statistical analysis. For the one-way repeated measures ANOVA, sphericity was tested by Mauchly's test and, when it was violated the ANOVA was corrected by the Greenhouse and Geisser test.

As the prednisone dose data over time were not normally distributed, a Friedman test was performed to determine whether there was a statistically significant difference in prednisone dose throughout a 26-week intervention. Pairwise comparisons were made using a Bonferroni correction for multiple comparisons.

To compare the reduction in clinical scores from baseline and the frequency of MG-ADL and QMG responders with the demographic and clinical characteristics of patients, we used the unpaired t-test (or Mann––Whitney U test in case of non-normally distributed data and/or presence of outliers and/or unequal variance as assessed by the Levene test) and the chi-squared test, respectively.

Significance was set at 0.05, two-tailed. Statistical analysis was performed using SPSS version 29.0.1.0 running on MACOS 12.6.6.

### Ethical Approval

All participating centres obtained written consent from each patient for the use of anonymized clinical data for research purposes. Following the requirements of the EAP, treatment with ravulizumab could only be initiated after approval by the local ethics committees at each center.

## Results

### Patients’ demographic and clinical characteristics at baseline

A total of 24 gMG patients, 10 females and 14 males, aged between 24 and 82 years (Median 60.5, IQR 52.5–67.5), attending the EAP for ravulizumab in Italy between March 2023 and December 2024, were enrolled in the study. The main demographic and clinical characteristics of single patients are summarized in Table 1. At study entry, disease duration ranged from 4 months to 35 years (median 5 yrs., IQR: 2.5–7.6); age at disease onset ranged from 18 to 81 years (median 53, IQR 38.5–61.5), with 8 patients (33.3%) before 50 years and 16 (66.7%) after 50 years. The worst MGFA level was IIB in 2 patients, IIIA in 1, IIIB in 5, IVB in 7 and V in 9, and between 1 and 5 myasthenic crises had been recorded in 11 patients. MGFA classification at baseline was IIb in 5 patients, IIIa in 5, IIIb in 12 patients, and IVb in 2 patients. Follow-up duration regarding ravulizumab treatment efficacy (evaluated through clinical scales) ranged from 2 to 74 weeks (median 26, IQR 26–42), considering two patients who discontinued treatment after 2 weeks (2nd infusion) and one patient who discontinued after 26 weeks (5th infusion).

**Table 1:** Main baseline demographic and clinical characteristic of patients enrolled in the EAP program

Patient	Sex	Age	Age at onset	Disease duration (M)	FU duration (M)	Thymectomy	Thymoma	MGFA BL	Weight	Comorbidities
1	F	57	37	224	15	Yes	Yes	IIIA	63	Diverticolitis
2	M	72	54	196	19	No	No	IIIB	95	AH, OSAS, ostheoporosis
3	F	24	18	53	15	Yes	Yes	IIIB	80	None
4	M	48	28	229	10	Yes	Yes	IIB	65	None
5	m	66	60	62	10	No	No	IIB	99	DM II
6	m	57	50	75	6 (w)	Yes	Yes	IIIA	85	Myelodisplastic syndrome
7	F	79	72	73	6	No	No	IIIA	75	AH, HF, obesity, headache
8	M	65	62	27	6	No	No	IVB	94	OSAS
9	M	60	52	87	6	No	No	IIB	85	None
10	M	34	29	54	6	No	No	IIB	77	None
11	F	64	28	425	4	No	No	IIB	60	AH, osteoporosis
12	M	82	81	4	1 (w)	No	No	IVB	95	Axonal polyneuropathy
13	F	40	35	43	7 (w)	Yes	No (thymic hyperplasia)	IIIA	61	Stiff Person syndrome
14	F	61	54	92	2	Yes	Yes	IIIB	72	None
15	M	71	69	27	2	Yes	Yes	IIIB	60	None
16	M	70	64	62	8	Yes	Yes	IIIB	79	DM II; AH; ICM; dyslipidemia
17	F	58	53	49	9	Yes	Yes	IIIB	62	DM II
18	F	46	43	17	12	Yes	Yes	IIIB	70	None
19	F	62	51	125	12	Yes	Yes	IIIB	63	None
20	M	54	53	5	9	Yes	Yes	IIIB	60	Autoimmune polyneuropathy
21	F	52	45	78	6	Yes	Yes	IIIA	65	None
22	M	65	62	29	6	No	No	IIIB	110	None
23	M	68	60	89	6	Yes	Yes	IIIB	86	Psoriasis, AH, diverticolitis, OSAS, HBV, osteoporosis
24	M	60	56	36	6	Yes	Yes	IIIB	99	AH

For safety concerns, clinical follow-up was continued throughout the study period also for patient who were discontinued from treatment.

### Changes in clinical scale scores during follow-up

The number of patients evaluated at each time point, the mean values of the MG-ADL and QMG at baseline and their change during the follow-up, and the number of MG-ADL and QMG responders are shown in Table 2.

**Table 2:** MG-ADL and QMG baseline mean values and differences of the means during a 26-week follow-up

	Baseline	Week 2	Week 10	Week 18	Week 26
MG-ADL§	9.37	−4	−5.29	−5.91	−5.58
(SD)	−3.28	−2.34	−2.86	−2.56	−2.96
95%CI	7.99–10.76	−4.98; −3.01	−6.50; −4.08	−7.00; −4.83	−6.83; −4.33
Median (IQR)	8.0 (6.0;12.0)	−3.0 (−5.75; −2.00)	−5.0 (−6.0; −4.0)	−6.0 (−7.5; −4.25)	−5.5 (−7.75; −3.25)
MG-ADL responders ^		17/24	19/24	17/22	17/21
(%)		−70.8	−79.2	−77.3	−81
QMG§	14.75	−4.5	−5.75	−6.21	−6.62
(SD)	−4.64	−2.99	−2.95	−3.23	−3.63
95%CI	12.79–16.71	−5.76; −3.23	−6.99; −4.50	−7.57; −4.84	−8.15; −5.09
Median (IQR)	14.5 (11.25–17.0)	−4.5 (−7.0; −2.25)	−5.5 (−8.0; −3.25)	−6.0 (−8.8; −4.0)	−7.0 (−8.0; −4.0)
QMG responders ^		Dec-24	15/24	14/22	14/21
(%)		−50	−62.5	−63.6	−66.7

Note: Values are expressed as mean ± SD and 95% CI

^§^ Mean score at baseline and mean difference with respect to baseline during follow-up

° Mean dosage at baseline and mean dosage change during follow-up

*MG-ADL *Myasthenia Gravis Activity of Daily Living, *QMG* Quantitative Myasthenia Gravis, *MGC* Myasthenia Gravis Composite

^ MG-ADL responders: patients with reduction in MG-ADL score of at least 3 points from baseline; QMG responders: patients with reduction in QMG score of at least 5 points from baseline

#### MG-ADL score

The percentage of MG-ADL responder patients (with a reduction ≥ 3 in MG-ADL score) was 70.8% at week 2, increasing to 79.2% at week 10 and 77.3% at week 18, with a shift to 81% at week 26. Among the non-responders, one patient discontinued treatment after week 26.

**Fig. 1:**
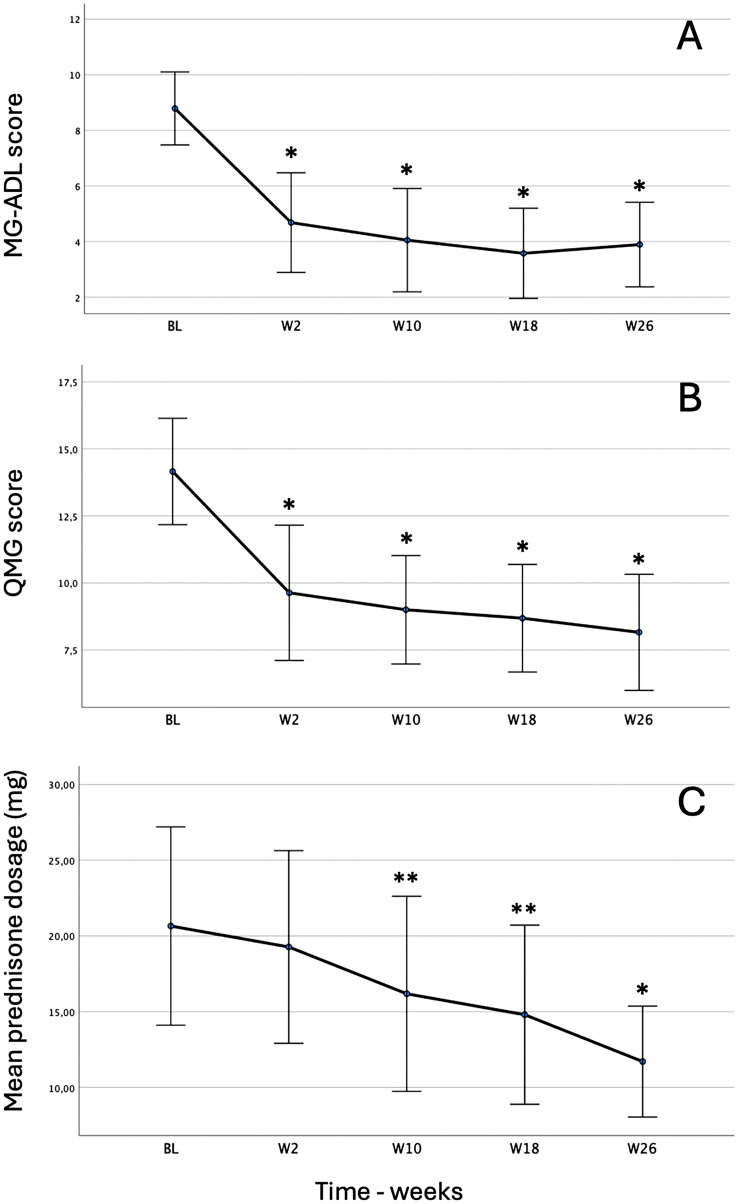
MG-ADL, QMG and mean prednisone daily dose changes during the first 26 weeks of Ravulizumab treatment in patients with anti-AChR gMG. *MG-ADL:* Myasthenia Gravis Activities of Daily Living scale. *QMG* Myasthenia Gravis quantitative scale. *W* weeks. *p < 0.001. **p < 0.05. Error bars show the standard error mean

As MG-ADL scores presented an outlier at weeks 18 and 26 and were not normally distributed, we used the non-parametric Friedman test for the statistical analysis of this variable. The test showed that MG-ADL score was significantly different at the different time points during ravulizumab (χ^2^(4) = 61.16; *p* < 0.001). Pairwise comparisons with a Bonferroni correction showed that MG-ADL score was significantly decreased from baseline (Median 8.0; IQR 6.0–12.0) to week 2 (Median 4.5; IQR 1.25–9.75; *p* < 0.001), week 10 (Median 3.50; IQR 1.25–6.75; *p* < 0.001), week 18 (Median 3.275; IQR 1.0–5.5; *p* < 0.001) and week 26 (Median 3.895; IQR 1.5–5.0; *p* < 0.001) but not from week 2 to week 10, 18 and 26 (Fig. 1A).

#### QMG score

The percentage of QMG responder patients (with a reduction ≥ 5 in QMG score) was 50% at week 2, 62.5% at week 10 and 63.6% at weeks 18 and reached 66.7% at week 26.

The one-way ANOVA, corrected by Greenhouse and Geisser test for violation of sphericity, showed that during the 26-week follow-up, the intervention produced significant changes in QMG score over time, F (2.42–55.72) = 39.43, *p* < 0.001, with QMG score decreasing from 14.75 ± 4.63 before the intervention to 8.12 ± 4.17 at week 26. Post hoc analysis with a Bonferroni adjustment revealed a significant mean difference in QMG score from pre-intervention to week 2 [− 4.50 (95% CI:—6.40 to—2.60), *p* < 0.001], week 10 [− 5.75 (95% CI: – 7.62 to – 3.88), *p* < 0.001], week 18 [− 6.21 (95% CI:—8.25 to—4.16), *p* < 0.001] and week 26 [− 6.62 (95% CI: 8.93–4.32), *p* < 0.001], but not from week 2 to week 10, 18 and 26 (Fig. 1B).

#### MGC score and MSE

Not all centres assessed MGC, which was therefore available in 18/24 patients at baseline with a median of 14.5 (IQR 13.0–17.25). The median MCG decreased to 10 (IQR 6.0–16.0) at week 2 and to 6.5 (IQR 4.75–12.25; 4.75–10.50) at weeks 10–18, and to 5.0 (IQR 4.0–9.0) at week 26.

Along the follow-up, MSE was achieved in 6/24 (25%) patients since week 2 and was maintained at weeks 10, 18 and 26 in all but one patient who had a momentary clinical deterioration at week 26. At the last available follow-up 9/24 (37.5%) of patients had achieved MSE.

### Patients’ characteristics and changes in clinical scores

**Fig. 2:**
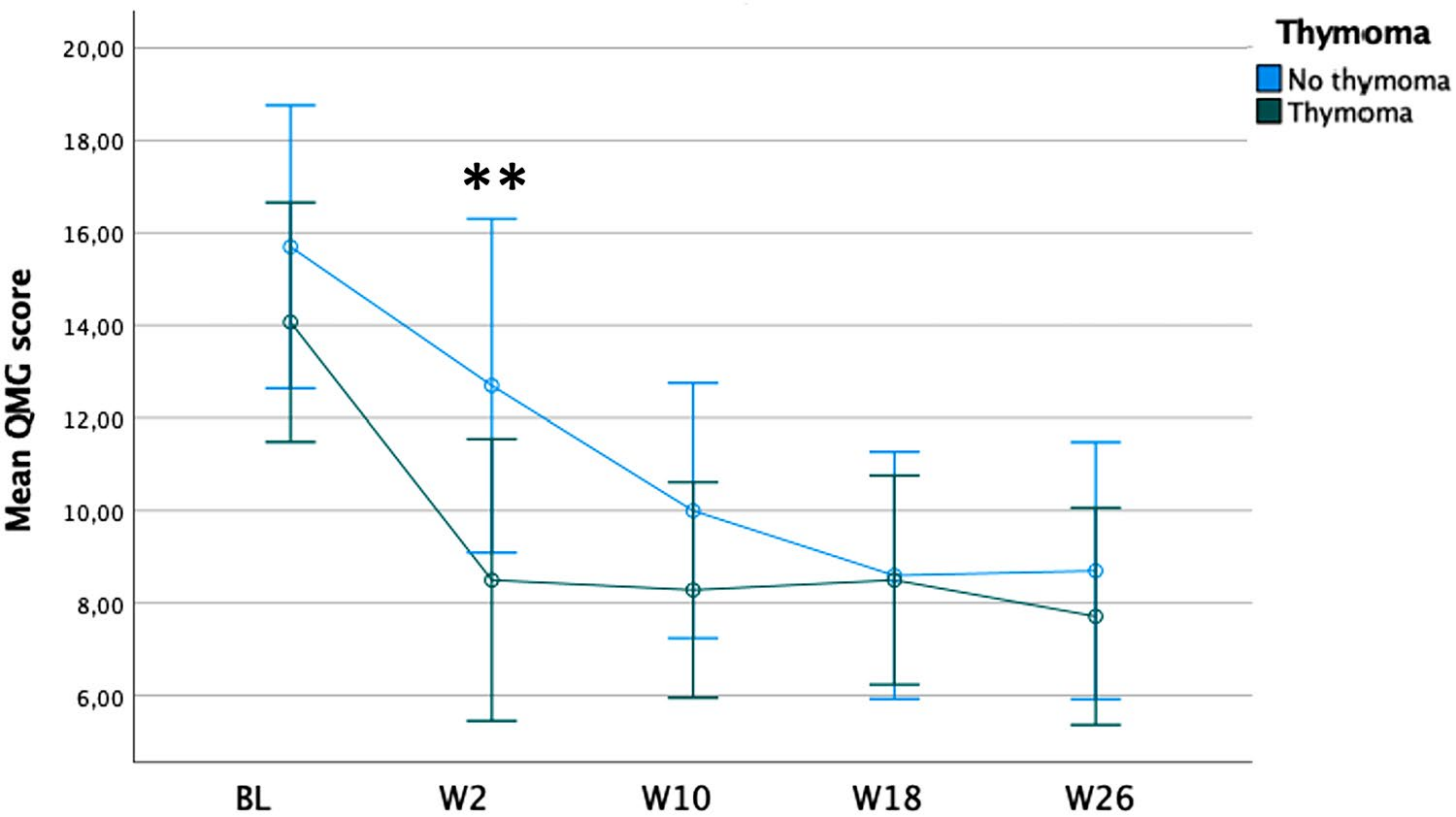
Mean QMG scores in thymoma-associated MG versus non-thymomatous MG patients. *p < 0.001. **p < 0.05. Error bars show the standard error mean

Univariate analysis showed that, among the demographic and clinical patients’ characteristics considered, thymoma was significantly associated with QMG score reduction and the rate of QMG responders at week 2. Specifically, QMG reduction was higher in thymoma-associated MG (TAMG) (−5.57 ± 2.95) than in non-TAMG patients (−3.00 ± 2.45), a statistically significant difference of -2.57 (95% CI, −4.94 to −0.20), t(22) = 2.251, p = 0.03. The frequency of QMG responders was observed in 71.4% of TAMG and 20% of non-TAMG patients [χ2(1) = 6.171, *p* = 0.036; odds ratio = 10.0, 95% CI = 1.44–69.26]. However, the comparison of reduction in QMG score was not significant at the other time points evaluated (Fig. 2). The Phi test showed that even this association was "strong" (φ = 0.507, *p* = 0.013) [12]. No other demographic or clinical characteristics were found to correlate with changes in scores on the rating scales.

### Prednisone reduction during follow-up

At baseline 22/24 (91.6%) patients were receiving steroids. During the follow-up period, a reduction in prednisone dosage was possible in 76% of patients, up to the withdrawal of the drug in 3 patients. Specifically, 33% of patients (7/21) decreased their prednisone dosage from 10 to 50% of the baseline dose (median 20%, IQR 13.33–28.57) at week 2; 57% (12/21) reduced their prednisone dosage from 9 to 100% (median 25.83%, IQR 20–52.85) at week 10; 72% (13/18) lowered prednisone from 4.35 to 100% (median 33%, IQR 23 0.63–60.00) at week 18; and 76% (13/17) lowered prednisone from 21.43 to 100% (median 46.66%, IQR 38.18–66.33) at week 26. Reduction of prednisone dosage was significant across the different time points during the intervention [χ^2^(4) = 37.440, *p* < 0.001]. Post hoc analysis revealed significant differences in prednisone dosage from pre-intervention (median 18.75 mg, IQR 13.75–26.25) to week 10 (median 15.00 mg, IQR 10–25, *p* = 0.013), week 18 (median 15.00 mg, IQR 10–20, *p* = 0.02), and week 26 *(*median 15.00 mg, IQR 8.12–17.50, *p* < 0.001). Similarly, significant differences were observed in prednisone dosage from week 2 (median 18.75 mgs, IQR 12.50–26.25) to week 18 (*p* = 0.013) and week 26 (*p* < 0.001) and from week 10 to week 26 (*p* = 0.039). No differences were found between pre-intervention and week 2, week 2 and week 10, week 10 and week 18 or week 18 and week 26 (Fig. 1C).

### Other therapies

Considering their past medical history, all but one patient had received at least one NSIST: azathioprine (n = 17), mycophenolate mofetil (n = 3), cyclosporine (n = 1), cyclophosphamide (n = 1) and rituximab (n = 3). In addition, 23/24 (95.8%) patients had received at least one IvIg cycle, and 9/24 (37.5%) had at least one PLEX. In the year prior to starting ravulizumab, 14/24 (58.3%) patients needed IvIg/PLEX treatment (8 IvIg, 1 PLEX, 5 both). None of the patients had previously been treated with anti-complement or anti-FcRn drugs.

At study entry 7/24 (29.2%) patients were on azathioprine, 3/24 (12.5%) were on mycophenolate mofetil, while 14/24 (58.3%) were not receiving NSIST.

During the study period, one patient was able to reduce the daily dose of azathioprine and another was able to reduce the daily dose of mycophenolate mofetil. No patient was completely withdrawn from NSIST. One patient, who had received 9 PLEX in the year prior to starting ravulizumab, underwent 2 PLEX and had to temporarily increase the daily steroid dose (see below).

### Thymectomy

Fifteen patients (62.5%) had previously undergone thymectomy, 14 (93.3%) for thymoma and one (6.7%) for thymic lymphoid follicular hyperplasia. Thymectomy was performed prior to the start of ravulizumab therapy in all patients except one with thymoma, who received complement inhibition therapy one month before thymectomy, due to an uncontrolled MG-related condition and lack of response to steroids, RTX and rescue therapies (IvIg and PLEX), which precluded the patient from undergoing the surgery.

Among all thymectomized patients, the interval between thymectomy and ravulizumab initiation ranged from 0 to 18 years, with a median of 4 years (IQR: 2–7 years). In the TAMG subgroup, the interval ranged from 0 to 18 years, with a median of 4.5 years (IQR: 2–7 years).

### Adverse events and withdrawals

One patient experienced an exacerbation of myasthenic symptoms without any apparent trigger at week 38, which was managed with two PLEX (with a subsequent supplementary dose of ravulizumab) and an increase in the daily steroid dosage from 15 to 37.5 mg daily. The patient continued treatment with ravulizumab, and by the last available follow-up (week 58) demonstrated good symptom control and was able to taper the daily steroid dosage back to 15 mg.

During follow-up 5 infectious adverse events were recorded. One patient experienced bronchitis, another had cystitis, one patient contracted pneumonia, one patient experienced herpes zoster and a fifth patient was diagnosed with iridocyclitis, which was probably associated to a toxoplasma infection. All adverse events resolved with appropriate therapy. The latter patient is currently undergoing specific treatment for ocular toxoplasma.

One patient developed arthralgias and joint effusions, with a diagnosis of psoriatic arthritis, which was probably triggered by the reduction of steroid therapy (from 25 to 5 mg daily on the 6th infusion, week 34), after approximately 16 years of prednisone at a dose of over 20 mg daily.

Three patients (12.5%) discontinued treatment with ravulizumab: the patient with iridocyclitis mentioned above who had no clinical benefit from ravulizumab decided to discontinue treatment. A second patient developed Steven-Johnson syndrome after the second infusion of ravulizumab. The third patient, who had a mild and unsatisfactory improvement, decided to discontinue treatment.

## Discussion

The EAP facilitated the evaluation of the efficacy and tolerability of ravulizumab in 24 patients with generalized anti-AChR gMG, in a real-world setting. Our study, similarly to the experimental ones [6, 7], has shown that in clinical practice ravulizumab leads to an improvement in clinical scores in patients with gMG, that this improvement is rapid, within the first two weeks, and remains stable over the following six months, and that it also allows a significant reduction in steroid therapy.

To our knowledge, apart from the registrational studies (CHAMPION trial and its OLE), that examined 175 and 169 patients respectively, three real-world studies have been published on the use of ravulizumab in gMG [13–15]. The first is a retrospective multicenter study of 18 gMG patients, 10 of whom were anti-complement naïve; the second is a prospective, exploratory study in 48 gMG anti-complement naïve; the third is a retrospective, comparison study between anti-complement and anti-FcRn drugs describing a cohort of 80 patients treated with ravulizumab.

In most of these studies, the patients presented with clinical scores at enrolment that did not differ from those in our sample, in particular concerning MG-ADL and QMG scores; as expected, the MG-ADL score in the study from Katyal et al. was lower than in our patients (10/18 patients were already under eculizumab treatment). However, looking at the comparable assessment points during follow-up, we found a greater reduction in both MG-ADL and QMG scores in our sample than in the others, as well as different proportions of MG-ADL and QMG responders and patients reaching MSE.

Looking at the change in clinical scores over time, we found that the most significant improvement in both MG-ADL and QMG occurred within the first 2 weeks of treatment, with the achievement of MSE state in a quarter of patients, followed by a stabilization of the response over the following months. This is a notable point in clinical practice and reflects the rapid action of ravulizumab, similar to other anti-complement agents such as eculizumab and zilucoplan [16, 17].

This early response to ravulizumab treatment was also reported in the CHAMPION trial and its OLE, and, similarly to our study, patients continued to reduce their MG-ADL and QMG scores over the following weeks, although their final improvement (evaluated at week 26) was less than in our sample. Though the differences in the design of the aforementioned studies call for caution when comparing the results, the difference in the variation of the scores on the rating scales at different time points is evident, which, apart from possible biases due to methodological differences, leads us to hypothesize a biological influence of factors specific to the study population.

In this context, the available demographic and clinical data of the studied populations compared with ours, showed only slight differences with those described from Stascheit et al. as a lower rate of male patients, a longer mean disease duration, a higher frequency of MGFA II at baseline, a lower frequency of thymoma (19% vs. 58% in our study), although with a similar frequency of thymectomy (61% vs. 62.5%); comparing with population described by Huntemann et al., we found differences only in the frequency of thymoma (15% vs. 58%), as well as with population described by Katyal et al. Finally, comparing to the CHAMPION trial, we only found a lower frequency of MGFA III patients in the trial population (47% vs. 71%).

On this basis we performed statistical analysis, which showed that thymoma was the only one of the above features to correlate significantly with both the reduction in QMG score and the frequency of QMG responders at the early phase of the study (week 2), leading us to hypothesize that the high frequency of thymoma in our sample, a peculiarity compared to the other studies, may have influenced this figure. Unfortunately, these data cannot be compared with larger samples, such as those from CHAMPION and its OLE study, which do not report information on thymectomy and thymoma. In any case, although TAMG is often characterized by more severe symptoms and a relatively poor prognosis, the access of TAMG patients to clinical trials with anti-complement drugs has been completely excluded, as in REGAIN trial, or severely restricted, as in the CHAMPION and RAISE trial [7, 16, 17]. However, in addition to some case reports [18, 19], a multicenter real-world cohort study of 22 TAMG patients reported a more significant improvement in MG-ADL and a faster and more frequent achievement of the MG-ADL responder rate [20] than in the REGAIN study [16] and in the other real-life experiences with anti-complement drugs [21–23], a figure which was not different from that of our study. This difference was attributed by the authors to a more severe disease status of TAMG patients at baseline. Other real-world studies reported a greater reduction in clinical scales and/or faster efficacy of eculizumab in patients with high MG-ADL and/or MGFA status at baseline comparing their data to REGAIN ones, and interestingly, the prevalence of thymoma was relatively high in those populations [24, 25].

In our study, both MG-ADL and QMG did not show any differences at baseline comparing patients with and without thymoma (8.42 ± 2.82 in TAMG vs. 10.70 ± 3.56 in non-TAMG and 14.07 ± 4.17 vs. 15.70 ± 5.29, respectively).

We also evaluated the potential influence of thymectomy on clinical response in the TAMG subgroup. The interval between thymectomy and the initiation of ravulizumab ranged from 0 to 18 years with a median of 4.5 years. All but one patient underwent thymectomy prior to starting ravulizumab; the latter underwent the procedure one month after treatment initiation due to precarious clinical condition that contraindicated surgery.

To date, data on the long-term effects of thymectomy in gMG patients remain limited. In the MGTX trial and its two-year extension phase [26, 27], the therapeutic effect of thymectomy on non-thymomatous gMG patients, was generally evident over a 36-month period post-surgery and tended to persist for up to 58 months, in terms of improvement in QMG scale and steroid-sparing effect. Two recent studies in patients with thymomatous gMG have shown overall clinical improvement after thymectomy, as assessed by the MG post-intervention scale (MG-PIS). In particular, clinical stable remission and improvement incidence at 5 and 10 years after thymectomy were reported in 18% and 36%, and 84% and 92% respectively reported, and MGFA class at symptom onset was identified as a predictor of worse outcome. [28] In the second study, after a mean follow-up period of 35.6 ± 25.7 months, 60% of patients showed an improvement in their gMG symptoms while 40% remained clinically stable. [29]

Given the median interval of 4.5 years between thymectomy and initiation of ravulizumab in our cohort, any clinical benefit from thymectomy should have already manifested or plateaued. Notably, we observed a significant improvement in the QMG scale as early as two weeks after initiation of ravulizumab, even in non-thymectomized patients, suggesting that the response would be more directly attributable to complement inhibition than to prior surgery. In addition, clinical severity as assessed by the MGFA clinical classification at disease onset and at baseline did not show any correlations with changes in scores on the rating scales.

In the single patient who underwent thymectomy one month after initiating ravulizumab treatment, a meaningful clinical improvement was observed by week 10. This temporal association further supports that the observed benefit could be attributable to the initiation of ravulizumab.

Although the relatively small number of patients in our study did not allow us to perform an ad hoc statistical analysis to understand whether thymoma was indeed independently associated with the more favourable outcome, these data lead us to hypothesise an influence of intrinsic biological factors related to thymoma. The complement system, particularly complement C3, plays a critical role in autoimmune diseases, and a decrease in complement C3 levels not only indicates increased disease activity but may also lead to an imbalance in immune regulation, exacerbating autoimmune responses [30]. Interestingly, TAMG patients with lower C3 levels are more likely to experience MG symptom progression and a worse prognosis after thymectomy than those with normal levels [31]. We could speculate that the lower level of C3 in TAMG could be an expression of its overconsumption, caused by a particularly predominant role of the complement cascade in the pathophysiology of NMJ dysfunction in TAMG patients. Comparing the expression of NMJ protective factors against complement attack, such as CD59, in TAMG and non-TAMG patients may help to clarify this issue [32].

Similarly to a post-hoc subgroup analysis of CHAMPION [33], we did not find any significant relationship between changes in clinical score and disease duration before ravulizumab start.

A significant steroid-sparing effect, allowing a reduction of daily prednisone dose in more than 70% of our patients, was in line with the available literature, even considering that a direct comparison is difficult due to different time points evaluated and variable clinical management [13, 15, 21–23]. In the first 6 months of follow-up, the average prednisone dose was nearly halved. Three patients were able to withdraw from steroids, and one patient discontinued NSIST. By the end of the follow-up period, 4 patients were receiving ravulizumab as their sole maintenance therapy.

Seven adverse events were recorded, the majority of which were infectious (5/6), all of which resolved with appropriate therapy. One patient is still undergoing treatment for suspected ocular toxoplasmosis, which has led to hypovision. Notably, one patient experienced Stevens-Johnson syndrome after the second infusion of ravulizumab, which was promptly treated with high-dose steroids and resolved. No reports of headache or diarrhea were noted. However, as adverse events were retrospectively collected using non-standardized forms, it is possible that some minor side effects may have been underreported.

### Limitations of the study

Several limitations deserve to be mentioned for this study. Firstly, the retrospective and real-world design of the study, based on medical records from seven different institutions, introduces inherent biases and inconsistencies, mainly due to the heterogeneity and small size of the sample. The study population included different subgroups of gMG patients varying in age, disease duration, and comorbidities.

In particular, the limited sample size constrained the scope of the analysis and reduced the statistical power of the findings. Furthermore, the high variability in ravulizumab treatment duration affected the consistency of the follow-up period, resulting in missing data and restricting the statistical analysis to the first 26 weeks.

The patient selection criteria also posed limitations. According to the EAP inclusion criteria, only individuals who had received steroids and at least one NSIST were eligible, while patients with treatment-refractory disease—typically eligible for eculizumab, but also in the CHAMPION trial—were excluded. This restriction may have introduced selection bias and limits the generalizability of the findings to the broader gMG population.

Additionally, not all patients were receiving steroids and/or the same type of immunosuppressive therapy at the time of enrolment. This variability, along with differences in treatment duration and follow-up, further contributed to the heterogeneity of the sample.

Lastly, inconsistencies in quality-of-life assessments across centers prevented standardized evaluation and precluded statistical analysis of this important outcome.

Prospective studies with larger cohorts are warranted to validate the potential benefits of ravulizumab, particularly in well-defined subgroups of gMG patients, and to more comprehensively assess outcomes such as long-term tapering or discontinuation of steroids and NSIST.

## Conclusions

Ravulizumab demonstrated a rapid, efficacious, and well-tolerated profile in our cohort of adult patients with anti-AChR antibody-positive generalized myasthenia gravis, further consolidating the results observed in the CHAMPION trial and other clinical studies conducted in real-world settings, showing particular early effectiveness in TAMG patients. Given the increasing (and costly) therapeutic options available for gMG, it is crucial to identify the patient groups that would benefit most from complement inhibition. Larger, and with longer follow-up, real-world studies are needed to further evaluate the benefits of Ravulizumab in gMG.

## Data Availability

Raw data that support the findings of the study are available from the corresponding author, upon reasonable request.
